# Knowledge and Attitude Toward Strabismus in Western Province, Saudi Arabia

**DOI:** 10.7759/cureus.6571

**Published:** 2020-01-05

**Authors:** Mona S Khojah, Sarah Al-Ghamdi, Shahad Alaydarous, Jumanah J Homsi, Ahmed Alhasan, Sara Alsubaie, Nizar Alhibshi

**Affiliations:** 1 Epidemiology and Public Health, King Abdulaziz University, Jeddah, SAU; 2 Epidemiology and Public Health, University of Jeddah, Jeddah, SAU; 3 Medicine, AlMaarefa University, Riyadh, SAU; 4 Ophthalmology, King Abdulaziz University Hospital, Jeddah, SAU

**Keywords:** strabismus, subsequent, knowledge, attitude

## Abstract

Background

Strabismus is a common eye condition having a potential subsequent impact on the psychological and socioeconomic domains of individuals suffering from strabismus. Therefore, this study aimed to find out the level of knowledge and treatability of strabismus in the western province of Saudi Arabia.

Methods

An observational cross-sectional study was conducted in 2018 among people who live in the western region of Saudi Arabia and were age 16 and above by using an online self-administered questionnaire.

Results

Out of 589 participants, 52.8% reported the correct definition of strabismus. The majority of responders agreed that strabismus is treatable (71.5%). In addition, a statically significant relation was found between knowledge of strabismus treatability and age, gender, work state, and level of education. Most participants were aware of the risk factors and complications of strabismus.

Conclusion

Our study found that the majority of participants had good knowledge of the definition, treatment, and complications of untreated strabismus. Participant’s age, education level, work state, and income were the main factors found to be significantly associated with knowledge of strabismus treatment options.

## Introduction

Strabismus is an ocular condition affecting the alignment of the visual axis, whether caused by abnormalities in binocular vision or anomalies of neuromuscular control of ocular motility [1]. It is commonly called by different names: squint, crossed eyes, deviating eyes, walleyes, goggle eyes, and wandering eyes [2]. The onset of strabismus may vary significantly [3]. The most squints occur in young children [4]. A 2017 study by Torp-Pedersen et al. showed that the prevalence of strabismus at seven years among nearly 100,000 children was 2.56% [5]. A recent study conducted in 2018 in Arar, Northern Saudi Arabia, reported strabismus in 14.7% of the studied sample [6].

Amblyopia, loss of vision, and cosmetic stigma are some of the consequences of untreated strabismus [7]. Some studies showed that strabismus is one of the common eye conditions having a potential subsequent impact on the psychological and socioeconomic domains later in the life of individuals suffering from strabismus [8-10], as well as on the effects on their self-image and interpersonal relationships with others, and plays a major role in selecting a partner for adults and in selecting a playmate for children [11-13].

Treatment options usually involve glasses, patching the good eye in case of amblyopia to force the use of the affected eye and surgery to correct the appearance of a squint [14]. Unfortunately, poor parental knowledge about strabismus adversely affects the early presentation and management of the child suffering from strabismus [15].

A study conducted in the Eastern province of Saudi Arabia emphasized the importance of public education and the early detection and management of strabismus to improve the educational opportunities and quality of life of these patients [16].

However, in developing countries like Saudi Arabia, there is a paucity of studies that aim to assess the level of knowledge, attitude, and practice of the general community concerning strabismus. Thus, we conducted this study to find out the level of knowledge and attitude towards strabismus in the Western province of Saudi Arabia.

## Materials and methods

This is an observational, descriptive, cross-sectional study approved by the institutional review board (IRB) of King Abdulaziz University. The study was conducted in 2018 among people who live in Jeddah city, in the western region of Saudi Arabia, by using an online self-administered questionnaire (a Google form) with close-ended questions. The questionnaire was validated by a panel of ophthalmology experts and distributed via a social media platform that is commonly used by the Saudi community such as Twitter, WhatsApp, and others. It was available between June 7, 2018, and June 17, 2018, and filled by the people who agreed to be involved in the study voluntarily. However, we included only participants who were aged 16 and above and living in Jeddah. Those who are medical students and workers or unable to answer the questionnaire due to a language barrier were excluded. The sample size obtained after considering the previous criteria was 589 participants. Their confidentiality was insured, as there was no personal information or identifier collection in the questionnaire.

The survey was composed of multiple-choice questions arranged into two sections. The first one collected demographic data such as age, sex, nationality, educational level, marital status, and economic level. The second section estimated the level of knowledge about strabismus through a number of questions dealing with the definition, risk factors, treatment options, and consequences of the disease on the lifestyle of the patient.

The analysis of the data was done using SPSS (version 25; IBM Corp., Armonk, NY). Qualitative variables were classified according to the aim of the study and then their frequencies and percentages were calculated. To evaluate the relationships between the variables, we used the chi-square test considering that a p-value of <0.05 was significant.

## Results

Out of 589 responders to the self-administered electronic questionnaire regarding the community's knowledge of strabismus, 107 (18.2%) were males and 482 (81.8%) were females (Table 1). In addition, 42.4% were younger than 30 years of age and 90.8% were Saudi. Regarding socioeconomic status, 63.3% were married, 78.1% were highly educated, 43.3% were office workers, and 45.7% were from the medium-income class.

**Table 1:** Demographic data of survey responders

Variable		Frequency	Percent (%)
Age	16-30 years	250	42.4
	31-45 years	215	36.5
	46 years and older	124	21.1
Gender	Male	107	18.2
	Female	482	81.8
Nationality	Saudi	535	90.8
	Non-Saudi	54	9.2
Marital Status	Married	373	63.3
	Single	183	31.1
	Divorced	25	4.2
	Widowed	8	1.4
Educational Level	Illiterate	4	.7
	Diploma or less	125	21.2
	Bachelor or more	460	78.1
Work	Office worker	255	43.3
	Free worker	12	2.0
	Housemaid	107	18.2
	Retired	36	6.1
	Student	139	23.6
	None	40	6.8
Income	Low income (<5000 SAR)	139	23.6
	Medium income (5000-10000 SAR)	269	45.7
	hHgh income (>10000 SAR)	180	30.6
Missing		1	0.2
Total		589	100.0

Among responders, only 38 (6.5%) had a child of their own who was diagnosed with strabismus while 37 (6.3%) had a child who was treated for strabismus. However, 224 (38%) of them admitted to having a relative with strabismus (Table 2).

**Table 2:** Social experience with strabismus among survey responders

		Frequency	Percent (%)
A child who is diagnosed with a squint	Yes	38	6.5
	No	368	62.5
	I do not know	14	2.4
	not applied	168	28.5
A child who is treated for a squint	Yes	37	6.3
	No	388	65.9
	I do not know	5	.8
	Not applied	158	26.8
A family member who has a squint	Yes	224	38.0
	No	316	53.7
	I do not know	48	8.1
Missing		1	.2
Total		589	100.0

In assessing knowledge of the strabismus definition, 52.8% of responders chose eye deviation as a definition while 33.4% chose to define it as abnormal eye movements (Table 3). However, there was no statistically significant relationship with gender, age, nationality, education, or socioeconomic state.

**Table 3:** Knowledge of strabismus definition among survey responders

		Frequency	Percent (%)
Knowledge of squint definition	Deviation of eye/s	311	52.8
	Abnormal movement of eye/s	197	33.4
	I do not know	52	8.8
	Deviation and abnormal movement of eye/s	28	4.8
	Total	588	99.8
Missing		1	.2

Regarding knowledge of strabismus treatment, the majority of responders agreed that strabismus is treatable, with 71.5% choosing yes (Table 4). Importantly, eye surgery, eye lenses, and eye patches were all chosen as possible treatment options, with 14.9%, 11.2%, and 5.4%, respectively (Table 5). However, 37% thought that all choices are possible treatment options for strabismus. In addition, a statically significant relation was found between knowledge of strabismus treatability and age, gender, and work state (p=000, 042, and 045, respectively). In the same way, a statically significant relation was found between knowledge of strabismus treatment options and age, educational level, work state, and income (p=001, 011, 017, and 002, respectively).

**Table 4:** Knowledge of the treatability of strabismus among survey responders

		Frequency	Percent (%)
Knowledge of the treatability of a squint	Yes	421	71.5
	No	30	5.1
	I do not know	137	23.3
	Total	588	99.8
Missing		1	.2

**Table 5:** Knowledge of treatment options for strabismus among survey responders

		Frequency	Percent (%)
Knowledge of treatment options for squint	Glasses or contact lenses	66	11.2
	Eye patches	32	5.4
	Eye surgery	88	14.9
	Glasses and patches	9	1.5
	Glasses and surgery	25	4.2
	Eye patches and surgery	12	2.0
	All are choices	218	37.0
	I do not know	138	23.4
	Total	588	99.8
Missing		1	.2

Additionally, the most frequently reported risk factors to develop strabismus from the responders' point of view were family history (16%) and eye refractive errors (12.9%) (Table 6). From the responders' point of view too, frequent complications of untreated strabismus were visual loss (4.6%), cosmetic stigma (3.9%), and poor self-image (2.4%); however, a clear majority chose "All of the above" with 55.2% (Table 7).

**Table 6:** Knowledge of risk factors to develop strabismus among survey responders

		Frequency	Percent (%)
Knowledge of risk factors to develop a squint	Family history	94	16.0
	Eye refractive errors (nearsightedness, farsightedness, and astigmatism)	76	12.9
	Systemic diseases (Down's syndrome, cerebral palsy...)	8	1.4
	Low socioeconomic state	0	0.0
	Smoker mother	0	0.0
	Family history, systemic diseases, low socioeconomic state, and smoker mother	1	.2
	Family history, systemic diseases, and smoker mothers	4	.7
	Eye refractive errors, systemic diseases, and smoker mothers	1	.2
	Family history, eye refractive errors, systemic diseases, and smoker mothers	3	.5
	Family history and smoker mother	1	.2
	Family history and eye refractive errors	46	7.8
	Family history and systemic diseases	25	4.2
	Eye refractive errors and systemic diseases	5	.8
	Family history, eye refractive errors and systemic diseases	33	5.6
	Family history, eye refractive errors and smoker mothers	2	.3
	systemic diseases and smoker mothers	1	.2
	All of the above	54	9.2
	Nothing of the above	25	4.2
	I do not know	210	35.7
	Total	589	100.0

**Table 7:** Knowledge of the complications of untreated strabismus among survey responders

		Frequency	Percent (%)
Knowledge of the complications of an untreated squint	Visual loss (partial or complete)	27	4.6
	Poor self-image	14	2.4
	Poor interpersonal relationships	5	.8
	Cosmetic stigma	23	3.9
	Poor self-image, interpersonal relationships, and appearance	23	3.9
	Visual loss, poor self-image, and interpersonal relationships	2	.3
	Visual loss and poor self-image	3	.5
	Poor self-image and interpersonal relationships	3	.5
	Visual loss and cosmetic stigma	10	1.7
	Poor self-image and appearance	11	1.9
	Visual loss, poor self-image, and appearance	3	.5
	Poor interpersonal relationships and appearance	10	1.7
	Visual loss, poor interpersonal relationships, and appearance	3	.5
	All of the above	325	55.2
	Nothing of the above	14	2.4
	I do not know	113	19.2
	Total	589	100.0

With respect to the community's awareness of strabismus as a health problem, 471 (80%) responders thought that strabismus treatment is a must for any age.

## Discussion

The study aimed to assess the knowledge of diagnosis and treatability of strabismus in the Western province, KSA. The identification and treatment of strabismus at an early age can lead to a better prognosis. Consequently, a lack of knowledge among parents and the population adversely affects the early diagnosis and management of strabismus [17].

The most obvious finding to emerge from the analysis is that the older workers with a good income and higher educational level have a better knowledge of strabismus treatability and treatment options. Also, 71.5% of the whole population knows that strabismus can be treated. This result is inconsistent with a study that took place in Nigeria, as 54% of the population did not know that strabismus can be treatment and only 21% knew about a medical treatment option. These results are likely related to the educational level where 22% of the Nigerian population in the previous study are illiterate [18].

The results of this study do not show any significant relationship between the knowledge of the definition of strabismus and gender, age, nationality, education, or socioeconomic status. This outcome is contrary to that of Isawumi et al., who found that women were more knowledgeable than men about strabismus, while there was no significant relationship to age [18]. This result likely related to the higher percentage of educated participants in our study (78.1%).

In the present study, most of the participants are aware of the risk factors of strabismus. The most frequently reported risk factors to develop strabismus are family history and eye refractive errors. However, other works of literature found that hereditary factors and ocular diseases are the most common risk factors for strabismus [18-19].

In our study, frequent complications of untreated strabismus are visual loss (4.6%), cosmetic stigma (3.9%), and poor self-image, and the majority chose "All of the above." Other studies showed that 95% of participants reported psychological complications regardless of the type of strabismus [20]. A cross-sectional study done in India found that children with strabismus had difficulty in making friends and finding a job [17]. The results reported by Ziaei et al. support this result; they found a positive improvement in the physical and psychological functions of children who were treated surgically for strabismus [21]. Additionally, strabismus had other vision-related complications, which could lead to an economic burden [19].

In this study, we faced several limitations regarding the distributed survey through different channels of social media. One of them was that the survey might not have been distributed enough to cover all the different social classes of the population. In addition, the study was restricted to the western region of Saudi Arabia; thus, it could not be filled by someone outside this region. Therefore, we recommend conducting the study among all Saudi Arabian residents.

## Conclusions

Our study found that more than half of the studied sample had good knowledge of the definition and complications of untreated strabismus, and the majority of participants agreed that strabismus is a treatable disease. Participant’s age, education level, work state, and income were the main factors found to be significantly associated with knowledge of the treatment options of strabismus. Overall, the level of awareness of strabismus was high and that is very important because previous knowledge of the disease may reduce the delays in seeking medical care, and this would reduce visual impairment and economic burden in the society. We recommend more public health education and to increase further awareness in all provinces of Saudi Arabia and to conduct other studies about amblyopia as a serious complication of untreated strabismus, which can lead to visual loss.
